# Associations of 6p21.3 Region with Age-related Macular Degeneration and Polypoidal Choroidal Vasculopathy

**DOI:** 10.1038/srep20914

**Published:** 2016-02-10

**Authors:** Zimeng Ye, Ping Shuai, Yaru Zhai, Fang Li, Lingxi Jiang, Fang Lu, Feng Wen, Lulin Huang, Dingding Zhang, Xiaoqi Liu, Ying Lin, Huaichao Luo, Houbin Zhang, Xianjun Zhu, Zhengzheng Wu, Zhenglin Yang, Bo Gong, Yi Shi

**Affiliations:** 1 Sichuan Provincial Key Laboratory for Human Disease Gene Study, School of Medicine, Sichuan Academy of Medical Sciences & Sichuan Provincial People’s Hospital, University of Electronic Science and Technology of China, Chengdu, China; 2 College of Life Science and Engineering, Southwest Jiaotong University, Chengdu, China; 3 Health Management Center, Sichuan Provincial People’s Hospital, Chengdu, China; 4 Department of ophthalmology, Sichuan Provincial People’s Hospital, Chengdu, China; 5 Zhongshan Ophthalmic Center, Guangzhou, China; 6 Clinical Medicine Department, Luzhou Medical College, Luzhou, China; 7 Sichuan Translational Medicine Hospital, Chinese Academy of Sciences, Chengdu, China

## Abstract

Neovascular age-related macular degeneration (AMD) and polypoidal choroidal vasculopathy (PCV) are leading causes of blindness in aging populations. This study was conducted to investigate the associations of chromosome 6p21.3 region, including *CFB-SKIV2L-TNXB-FKBPL-NOTCH4* genes, with both neovascular AMD and PCV. Six single nucleotide polymorphisms (SNPs) in this region and two known AMD-associated SNPs in *CFH* (rs800292) and *HTRA1* (rs11200638) were genotyped in a Han Chinese cohort composed of 490 neovascular AMD patients, 419 PCV patients and 1316 controls. Among the SNPs, *TNXB* rs12153855 and *FKBPL* rs9391734 conferred an increased susceptibility to neovascular AMD (*P* = 2.8 × 10^−4^ and 0.001, OR = 1.80 and 1.76, respectively), while *SKIV2L* exerted a protective effect on neovascular AMD (*P* = 2.2 × 10^−4^, OR = 0.49). Rs12153855C and rs9391734A alleles could further increase the susceptibility to AMD in subjects with rs800292, rs11200638 and rs429608 risk alleles. However, only the association of *SKIV2L* rs429608 remained significant after adjusting for rs800292, rs11200638 and the other 5 SNPs. The protective haplotype AATGAG exhibited significant association with neovascular AMD (permutation *P* = 0.015, OR = 0.34). None of the SNPs in this region was associated with PCV. Association profiles of 6p21.3 region showed discrepancy between neovascular AMD and PCV, indicating possible molecular and pathological differences between these two retinal disorders.

Age-related macular degeneration (AMD) is a leading cause of visual loss and legal blindness among the elderly people in both Eastern and Western populations^1^,^2^,^3^,^4^. Previous studies have revealed that early and late AMD prevalence rates are 4.7–9.5% and 0.2–1.0% in Han Chinese population, respectively^5^,^6^. In the early stage of AMD, the major pathological hallmark is the presence of large drusen, which is the accumulation of extracellular protein that build up between Bruch’s membrane and the retinal pigment epithelium (RPE). The later stages of this ocular disorder are characterized by geographic atrophy (GA) of the RPE and cone photoreceptors, or choroidal neovascularization (CNV). AMD can be categorized as ‘dry’ and ‘wet’ forms, both of which can compromise the central vision^7^. The cause of AMD is multifactorial, with smoking, age, and genetic background being the major risk factors^7^,^8^. Polypoidal choroidal vasculopathy (PCV) is a hemorrhagic and exudative macular disease, which is characterized by subretinal and intraretinal hemorrhage, orange retinal lesions, macular choroidal neovascularization, and sudden and painless visual loss^9^. The incidence of PCV in neovascular AMD patients is approximately 22–33% in Chinese population, which is relatively higher when compared with 10–13% in Caucasians^10^,^11^.

Clinically, it remains controversial as to whether PCV is a subtype of neovascular AMD or a separate disease entity^10^. Similarities between PCV and neovascular AMD have been observed in demography, pathology and manifestation, but differences have been noted in clinical features, histopathology and response to treatment^12^. While genetically, whether a true association exists between neovascular AMD and PCV is still poorly understood. The *high temperature required factor A1* gene (*HTRA1*) and the *complementary factor H gene* (*CFH*) were reported to be significantly associated with AMD in various populations, which were later replicated in PCV^13^. On the other hand, several AMD susceptible genes, including *cholesterylester transfer protein* (*CETP*), *superkiller viralicidic activity 2-like* (*SKIV2L*), *complement component 3* (*C3*), *elastin* (*ELN*), and *apolipoprotein E* (*APOE*) showed different association profiles between AMD and PCV^11^,^14^,^15^,^16^,^17^. These evidences, however, are neither sufficient nor cogent enough to interpret the relationship between neovascular AMD and PCV. It was, therefore, the purpose of this study to investigate whether the differences in clinical presentations between these two retinal disorders can be attributed to other genetic factors that may reveal different underlying pathogenic mechanisms.

*Complement component 2* (*C2*) and *complement factor B* (*CFB*) were paralogous genes located 500 bp from the major histocompatibility complex (MHC) class III region on chromosome 6p21^18^. C2 and CFB function as activators of classical and alternative complement cascade, respectively. Single nucleotide polymorphisms (SNPs) in the *C2* and *CFB* genes were reported to be associated with AMD, while these associations were inconsistent and inconclusive among different populations^19^,^20^,^21^,^22^,^23^. Recently, a genome-wide association study (GWAS) and several case-control association studies demonstrated that the *superkiller viralicidic activity 2-like* gene (*SKIV2L*), adjacent to the *C2-CFB* region, conferred a protective effect of AMD^15^,^21^,^24^. In our previous study, rs429608 in the *SKIV2L* gene has also been found to be associated with AMD^25^. SNPs in the *TNXB-FKBPL-NOTCH4* region, downstream of the *C2-CFB-SKIV2L* region, have been identified to be associated with AMD in the UK population through a GWAS^26^. More recently, the Genetics of AMD in Asians (GAMA) Consortium reported that a functional SNP rs12661281 (Asp47Val) in the *SLC44A4* gene, upstream of the *C2-CFB-SKIV2L* region, was associated with AMD in East Asians^27^. All of these SNPs, including rs12661281, rs541862, rs429608, rs12153855, rs9391734, rs2071277 and rs3132946, were located at chromosome 6p21.3 region. Taking these evidences together, it is possible that more genetic variants in this region may be associated with AMD and PCV.

Functionally, SKIV2L plays a role in the degradation of RNAs and autophagy, increased autophagy of RPE has been associated with drusen formation^28^,^29^,^30^. Tenascin-X, encoded by the *TNXB* gene was shown to involve in the collagen and elastin networks, both collagen and elastin are present in the Bruch’s membrane^31^,^32^. In addition, FKBPL has been linked to proangiogenic hypoxic signals, hypoxic stress in the micro-environment of the photoreceptor/RPE/Bruch’s membrane/choriocapillaris complex has been reported to be an underlying cause of pathophysiology of AMD^33^,^34^. Moreover, NOTCH4 is one of the four cell surface receptors of the Notch signaling pathway, Notch signaling involves in the development of retinal vasculature by regulating the specification of endothelial cells into stalk and tip cells^35^. As stated above, the functions of these genes could possibly lead to the relevance between the 6p21.3 region and neovascular AMD/PCV.

In the present study, we have genotyped these 6 SNPs in the 6p21.3 region (including rs541862, rs429608, rs12153855, rs9391734, rs2071277 and rs3132946) and 2 major AMD associated SNPs (rs800292 in *CFH* and rs11200638 in *HTRA1*) using SNaPshot method, and tested their associations with both neovascular AMD and PCV in a Han Chinese population composed of 490 neovascular AMD patients, 419 PCV patients and 1316 controls. As for rs12661281, since we have been part of the previous GAMA Consortium study, the AMD and PCV samples used in the GAMA Sichuan replication cohort were almost the same as the samples used in the present study, so we did not genotype rs12661281. Instead, we re-evaluated the data, because we have not stratified them into neovascular AMD and PCV sub-groups before.

## Results

### Association Study of the *CFB-SKIV2L-TNXB-FKBPL-NOTCH4* Region

As shown in Table 1, a total of 2225 subjects were recruited for genotyping, including 490 neovascular AMD patients, 419 PCV patients and 1316 unrelated controls. There were more males in case and control groups, therefore, we adjusted gender in the association studies using binary logistic regression. Notably, the mean age of control group was older than that of both case groups, this was due primarily to that we purposely recruited subjects older than 60 as controls to eliminate the confounding effects from younger subjects, thus age was not adjusted in the association analysis.

None of the 8 SNPs showed significant deviation from Hardy-Weinburg equilibrium in the three groups (*P* > 0.001, Supplementary Table 1). Allelic associations and minor allele frequencies were shown in Table 2. Two major variants, rs800292 and rs11200638, were significantly associated with both AMD and PCV as expected (Table 2). However, rs429608 (*P* = 2.2 × 10^−4^, OR = 0.49, 95% CI: 0.34–0.72), rs12153855 (*P* = 2.8 × 10^−4^, OR = 1.80, 95% CI: 1.31–2.48) and rs9391734 (*P* = 0.001, OR = 1.76, 95% CI: 1.27–2.50) showed significant association only with neovascular AMD, but not with PCV (Table 2). The minor allele of rs429608 (A) showed a protective effect for neovascular AMD, and this was consistent with our previous study^25^. Meanwhile, the minor allele of rs12153855 (C) and rs9391734 (A) conferred an increased risk of neovascular AMD. Rs541862 (*CFB*), rs3132934 (*NOTCH4*) and rs2071277 (*NOTCH4*) were neither associated with AMD nor with PCV in this study (Table 2). In addition, when we compared neovascular AMD with PCV, significant differences were observed in rs12153855 (*P* = 0.001, OR = 2.10, 95% CI: 1.35–3.26), rs9391734 (*P* = 0.001, OR = 2.33, 95% CI: 1.43–3.80) and rs11200638 (*P* = 0.001, OR = 1.40, 95% CI: 1.14–1.72). It was noteworthy that rs12153855 and rs9391734 appeared to be risk factors for neovascular AMD (OR = 1.80 and 1.76, respectively), but they showed a protective trend for PCV (OR = 0.84 and 0.87, respectively).

We further investigated the associations of these SNPs with AMD and PCV using 4 different genetic models (homo, hetero, dominant and recessive models) (Supplementary Tables 2 & 3). For rs429608 in the *SKIV2L* gene, significant associations were detected under hetero (*P* = 0.002, OR = 0.54, 95% CI: 0.37–0.79) and dominant (*P* = 0.001, OR = 0.52, 95% CI: 0.35–0.75) models (Table 3), suggesting subjects carrying rs429608 AA/AG genotypes were less likely to be suffered from neovascular AMD than those carrying GG genotype. For rs12153855 (*TNXB*) and rs9391734 (*FKBPL*), significant associations were revealed under hetero (*P* = 0.001, OR = 1.84, 95% CI: 1.31–2.60 and *P* = 0.002, OR = 1.78, 95% CI: 1.24–2.50, respectively) and dominant (*P* = 3.30 × 10^−4^, OR = 1.85, 95% CI: 1.32–2.59 and *P* = 0.001, OR = 1.80, 95% CI: 1.27–2.55, respectively.) models (Table 3), indicating rs12153855 CC/CG and rs9391734 AA/AG genotypes conferred increased risks of neovascular AMD. However, none of rs429608, rs12153855 and rs9391734 showed significant association under these genetic models in PCV (Supplementary Table 3).

The genetic effects of rs12153855 and rs9391734 were further evaluated under the allelic model in the context of rs11200638, rs800292 and rs429608. The stratification of rs11200638, rs800292 and rs429608 were defined by the dominant model with the risk allele as reference (Table 4). In the strata of rs11200638AA+AG, rs800292CC+CT and rs429608GG+AG genotypes, rs12153855C and rs9391734A alleles showed significantly increased risk effects on neovascular AMD (Table 4). In comparison, in the strata of rs11200638GG, rs800292TT and rs429608AA genotypes, neither rs12153855 nor rs9391734 showed significant association with neovascular AMD (*P* > 0.05). These data indicated that rs12153855C and rs9391734A alleles would further increase the susceptibility to neovascular AMD in subjects carrying rs11200638, rs800292 and rs429608 risk alleles.

Multiple logistic regression analysis was conducted with all of the 8 genotyped SNPs and gender as a covariate (Table 5). Only *SKIV2L* rs429608, in the *CFB-SKIV2L-TNXB-FKBPL-NOTCH4* region, remained significant association with neovascular AMD when conditioning on other covariates (*P* = 0.016, Table 5), indicating its independent effects on neovascular AMD.

**Figure 1:**
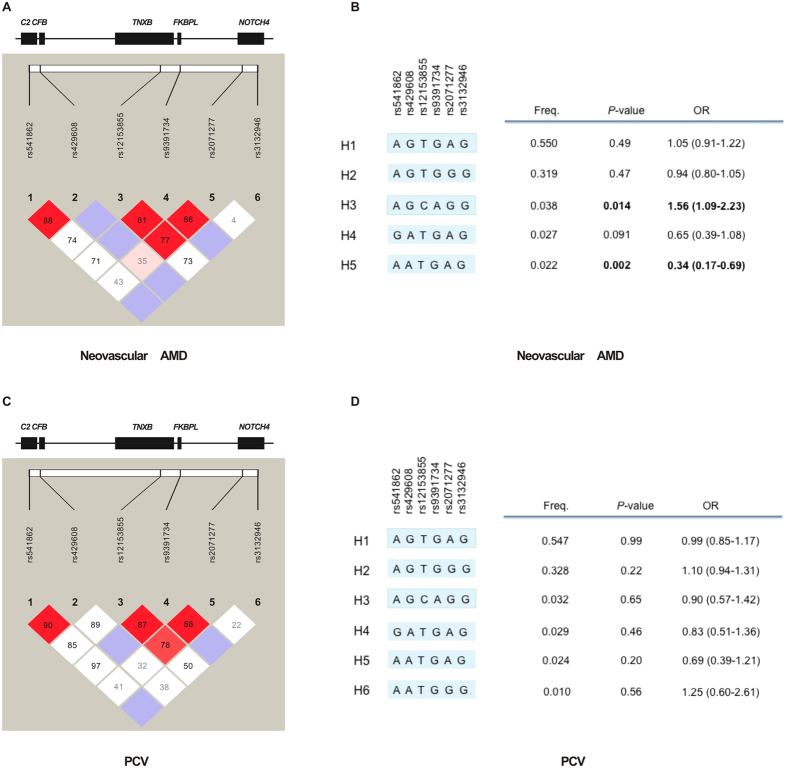
Linkage disequilibrium (LD) structure across *CFB-SKIV2L-TNXB-FKBPL-NOTCH4* region and results of haplotype-based association study (D’ values shown). (**A**) LD was measured using combined AMD case and normal control data. The physical position of each SNP is shown in the upper diagram. Each box provides estimated statistics of the coefficient of determination (D’), with darker shades representing stronger LD. (**B**) For AMD, 5 haplotypes were observed. AGCAGG conferred an increased susceptibility to AMD (*P* = 0.014, OR = 1.56), but it could not withstand permutation procedure (permutation *P* = 0.10). AATGAG showed a protective effect on AMD (*P* = 0.002, OR = 0.34), and it remained statistically significant after correction for multiple testing (permutation *P* = 0.015). (**C**) LD was measured using combined PCV case and normal control data. (**D**) For PCV, 6 haplotypes were observed, but no significant association was detected.

Finally, we analyzed the linkage disequilibrium (LD) structure across the *CFB-SKIV2L-TNXB-FKBPL-NOTCH4* region using the genotype data of these 6 SNPs in this region. LD values were displayed by D’ (Fig. 1A,C) and r^2^ (Supplementary Figure 1) scores, respectively. Haplotype-based associations were shown in Fig. 1B,D. Five haplotypes were observed between the neovascular AMD and control groups. The protective haplotype AATGAG and the risk haplotype AGCAGG showed significant association with neovascular AMD (*P* = 0.014, OR = 1.56, 95% CI: 1.09–2.23; *P* = 0.002, OR = 0.34, 95% CI: 0.17–0.69; respectively, Fig. 1B). However, only the protective haplotype AATGAG remained significant association after correction for multiple testing (permutation *P* = 0.015, permutation *P* = 0.10 for the risk haplotype AGCAGG). No significant association was found with PCV of the 6 observed haplotypes (Fig. 1D).

### Re-evaluation of the *SLC44A4* rs12661281

In the GAMA Consortium study, the researchers only conducted stratified analysis in the discovery samples (ref. 27 Supplementary Table 3). So in order to investigate whether rs12661281 showed significant association only with neovascular AMD/PCV, we stratified the subjects by sub-groups and re-evaluated the data of rs12661281 by using different genetic models (Supplementary Table 4). Interestingly, rs12661281 was also only associated with neovascular AMD, the association was observed under allelic model (*P* = 0.028, OR = 1.29, 95% CI: 1.02–1.62), homo model (*P* = 0.00066, OR = 4.21, 95% CI: 1.72–10.31) and recessive model (*P* = 0.00073, OR = 4.15, 95% CI: 1.70–10.14). But for PCV, no association was revealed under any of these genetic models (*P* > 0.05).

## Discussions

We conducted a comprehensive evaluation of the 6p21.3 region in both neovascular AMD and PCV. Two genetic variants in this region, *TNXB* rs12153855 and *FKBPL* rs9391734, are significantly associated with neovascular AMD in a Han Chinese population. As expected, associations were also observed for *CFH* rs800292 and *HTRA1* rs11200638 with both AMD and PCV, and for *SKIV2L* rs429608 with AMD.

CFB were expressed in the neural retina, RPE, choroid and Bruch’s membrane. Plasma concentration of CFB in AMD patients was significantly higher than that in normal controls^18^,^36^. Therefore, it is plausible that genetic variants of *CFB* gene may be associated with the susceptibility to AMD. Cipriani *et al.*^26^ reported that *CFB* rs541862 was significantly associated with AMD in the UK population through a genome-wide association study. In the same year, Nakata *et al.* noted that rs541862 was associated with typical AMD and PCV in a Japanese cohort^21^. Tanaka *et al.* took a step further, they categorized AMD patients into typical AMD, PCV and retinal angiomatous proliferation (RAP) groups, then categorized the PCV group into polypoidal CNV and typical PCV subgroups. They found that rs541862 was associated with polypoidal CNV but not with typical PCV, indicating that PCV might be genetically divided into polypoidal CNV and typical PCV^37^. In the current study, however, rs541862 neither associated with neovascular AMD nor with PCV in the Han Chinese cohort.

SKIV2L was thought to function as RNA helicase, involving exosome recruitment and activation. Also, SKIV2L was reported to play a role in the mechanism of autophagy, and increased autophagy of RPE was related to drusen formation^28^,^29^,^30^. Previously, *SKIV2L* rs429608 has been associated with AMD in US, UK and Han Chinese populations, all of these studies indicated the minor allele of rs429608 as a protective factor^24^,^25^,^26^. Liu *et al.* reported that rs429608 was only associated with neovascular AMD but not with PCV in a Chinese study group^15^. The results of this study were in accordance with the previous genetic studies by these researchers.

*TNXB* rs12153855 and *FKBPL* rs9391734 were highly linked. On the one hand, *TNXB* gene encodes tenascin-X, tenascin-X appears to control cell adhesion and migration, and also shows to involve in the maturation and maintenance of collagen and elastin networks^31^,^32^,^38^. Both collagen and elastin are present in the Bruch’s membrane, which could possibly lead to the relevance between *TNXB* gene and AMD. On the other hand, *FKBPL* encodes the FKBP-like protein, which plays an essential role in zebrafish and murine blood vessel development^33^. In human body, FKBPL is specifically downregulated by proangiogenic hypoxic signals^33^. Hypoxic RPE cells can produce angiogenic substances, such as vascular endothelial growth factor (VEGF), to stimulate the growth of new vessels from choriocapillaris complex, resulting in CNV^34^.In the original UK GWAS^26^, rs12153855 and rs9391734 were significantly associated with AMD, minor alleles of these two polymorphisms were risk factors. In the current study, both rs12153855 and rs9391734 only associated with neovascular AMD but not with PCV.

NOTCH 4 is a member of the Notch signaling pathway, which can mediate the development of retinal vasculature^35^. Notch signaling also contributed to angiogenic homeostasis by providing a counterbalance to proangiogenic pathways such as that mediated by VEGF^39^. In the UK GWAS^26^, *NOTCH4* rs2071277 was significantly associated with AMD; and rs3132934 showed nominal association in the discovery cohorts (P = 0.0027) but demonstrated no association in the English and Scottish replication cohorts^26^. As for the present study, our results suggested rs2071277 and rs3232934 were not associated with neovascular AMD or PCV.

In the current study, *SKIV2L* rs429608, *TNXB* rs12153855 and *FKBPL* rs9391734 presented different association profiles between neovascular AMD and PCV. These SNPs were significantly associated with neovascular AMD, but not with PCV. When we compared PCV with neovascular AMD by binary logistic regression, significant differences were showed in SNPs of rs429608, rs12153855, rs9391734 and rs11200638 (Table 2). It was noteworthy that rs12153855 and rs9391734 appeared to be risk factors for neovascular AMD (OR = 1.80 and 1.76, respectively), but they showed a protective trend for PCV (OR = 0.84 and 0.87, respectively). However, the minor allele frequencies of rs12153855 (0.042) and rs9391734 (0.041) were a little low in the Han Chinese population, thus their effect on genetic differentiation of neovascular AMD and PCV might be limited. Although risk effects of *HTRA1* rs11200638 were detected on both neovascular AMD and PCV, the effect was more pronounced in neovascular AMD than that in PCV (OR = 2.31 and 1.65, respectively). Furthermore, 2 haplotypes generated by the 6 SNPs of the *CFB-SKIV2L-TNXB-FKBPL-NOTCH4* region showed significant association with neovascular AMD. On the other hand, no haplotypes in this region were associated with PCV (Fig. 1).

In addition, we found a very interesting correlation between rs12661281 and AMD. According to the previous GAMA Consortium study^27^, *SLC44A4* gene has been found to be associated with AMD in East Asians through a genome-wide and exome-wide association study, and it may represent Asian-specific genetic association for AMD. The researchers conducted stratified analysis in the discovery samples (ref. 27 Supplementary Table 3). In the Hong Kong discovery cohorts, rs12661281 neither associated with typical neovascular AMD (tAMD) nor with PCV; in the Japan discovery cohort, rs12661281 associated with both tAMD and PCV; while in the Singapore discovery cohort, rs12661281 only associated with tAMD but not with PCV. Due to the potential importance to stratify the AMD samples into neovascular AMD and PCV, we have re-evaluated the data of the Sichuan replication cohort in the GAMA Consortium study. Similar to the results of the Singapore cohort, rs12661281 only associated with neovascular AMD in our samples (Sichuan Samples).

Taken the results of previous studies^15^,^25^,^26^ and this study together, it suggested that the chromosome 6p21.3 region was associated with neovascular AMD and this region might play an important role in pathogenisis of neovascular AMD in the Han Chinese population. Genetic variants in the *TNXB* and *FKBPL* genes would further increase the risk of neovascular AMD in subjects carrying *HTRA1* rs11200638, *CFH* rs800292 and *SKIV2L* rs429608 risk alleles. Association profiles of the 6p21.3 region showed discrepancy between neovascular AMD and PCV, indicating possible molecular and pathological differences between these two retinal disorders. The SNPs of rs12153855 and rs9391734 could be applied as biomarkers to differentiate the neovascular AMD and PCV. Further research for the effects of the 6p21.3 region on neovascular AMD and PCV will provide additional insights.

## Methods

### Study Participants

All study subjects were unrelated Han Chinese recruited from the ophthalmology clinic at Sichuan Provincial People’s Hospital. All participants underwent a standard ophthalmic examination protocol, including optical coherence tomography (OCT), indocyanine green angiography (ICGA), ocular tonometry, slit-lamp biomicroscopy, best-corrected visual acuity measurement, color fundus photographs, and fluorescein angiography. All AMD cases selected for this study had at least one eye affected by neovascular AMD. All PCV cases were diagnosed using ICGA. Individuals with other causes of CNV, or with both CNV and PCV lesions in the same or fellow eye, were excluded. All controls were matched by geographic area and were given complete ophthalmic examinations, they were included on the following criteria: 1) 60 years or older; 2) no signs of early AMD or macular degeneration of any cause; and 3) no other major eye diseases, except for mild senile cataracts or mild refractive errors.

For the association study on the *CFB-SKIV2L-TNXB-FKBPL-NOTCH4* region, a total number of 490 neovascular AMD patients, 419 PCV patients and 1316 normal matched controls were recruited. Characteristics of the study subjects were listed in Table 1. And for the re-evaluation of the *SLC44A4* rs12661281, the data were gained from the previous replication study in the GAMA Consortium research. The characteristics of the Sichuan replication cohort were reported in ref. 27.

This study was approved by the Ethnics Committee on Human Research of Sichuan Provincial People’s Hospital. All participants signed informed consent prior to participation in the study. The study procedures were performed in accordance with the tenets of the Declaration of Helsinki.

### SNP Selection and Genotyping

Previous GWAS in the UK population identified 5 novel SNPs in the chromosome 6p21.3 region, including rs541862 in *CFB*, rs12153855 in *TNXB* (*tenascin XB*), rs9391734 in *FKBPL* (*FK506 binding protein like*), rs2071277 and rs3132946 in *NOTCH4*^26^. These SNPs were chosen as candidate variants in the present study. Additionally, because rs429608 in *SKIV2L*, rs800292 in *CFH* and rs11200638 in *HTRA1* have been previously reported to be associated with AMD in Han Chinese, they were also included in this study. Venous blood of each subject was drawn and collected in an EDTA-containing tube. Genomic DNA was extracted from the blood using a Gentra Puregene Blood DNA kit (Gentra, Minneapolis, MN). SNP genotyping was conducted by the dye terminator-based SNaPshot method (Applied Biosystems, ABI, Foster City, CA), with success rate and accuracy greater than 99%, as judged by random re-genotyping of 10% of the samples in the subject group.

The genotype data for rs12661281 in *SLC44A4* were obtained from ref. 27. Because the neovascular AMD and PCV samples were highly overlapped between the present study and the previous replication study in the GAMA Consortium research, we did not genotype rs12661281 in this study. Instead, we re-evaluate the data by stratifying them into neovascular AMD and PCV sub-groups.

### Statistical Analysis

All statistical analyses were conducted using SPSS software version 17.0 (SPSS, Inc., Chicago, IL). Hardy-Weinberg equilibrium (HWE) of each SNP was tested with a standard observed-expected chi-square test (χ^2^ test). The genetic association analysis was carried out by constructing 2 × 3 tables of the genotype counts and 2 × 2 tables of the allele counts for each SNP in both patient and control groups. Subsequently, Pearson χ^2^ statistics were calculated and *P* values were computed by comparing the statistic to a χ^2^ distribution with 1 or 2 degrees of freedom for the allelic and genotypic tests. The odds ratios (ORs) and corresponding 95% confidence interval (CI) were estimated with the minor allele as reference, and were calculated using the χ^2^ test. The overall association study was adjusted for gender with binary logistic regression. For the significance threshold of *P* values, Bonferroni correction was applied to adjust the *P* values by the number of strata. In the overall allelic association study, as 8 SNPs were chosen, a *P* value of less than 0.00625 (0.05/8) was considered statistically significant.

The genetic effects of rs12153855 and rs9391734 were further evaluated under the allelic model in the context of rs11200638, rs800292 and rs429608. The stratifications of rs11200638, rs800292 and rs429608 were under the dominant model with the risk allele as reference. For Bonferroni correction, as 2 SNPs (rs12153855 and rs9391734) were analyzed under 6 strata (2 strata for each of rs11200638, rs800292 and rs429608), a *P* value less than 0.0042 [0.05/ (2 SNPs ×6 strata)] was considered statistically significant. Multiple logistic regression analysis was conducted under the allelic model using binary logistic regression, with all of the 8 SNPs and gender as a covariate.

Haplotype analysis was carried out with Haploview 4.2 software (Daly Lab at the Broad Institute, Cambridge, MA), in order to assess linkage disequilibrium (LD) patterns and haplotype association statistics. Haplotype blocks were defined using the “custom” option implemented in Haploview software, all of the 6 SNPs in the *CFB-SKIV2L-TNXB-FKBPL-NOTCH4* region were placed within 1 haplotype block. LD values were displayed in D’ and r^2^, respectively. To correct for multiple testing in the haplotype association analysis, 10,000 permutations were run using Haploview software. The OR and 95% CI for each haplotype were calculated using SPSS.

## Additional Information

**How to cite this article**: Ye, Z. *et al.* Associations of 6p21.3 Region with Age-related Macular Degeneration and Polypoidal Choroidal Vasculopathy. *Sci. Rep.* **6**, 20914; doi: 10.1038/srep20914 (2016).

## Supplementary Material

Supplementary Information

## Figures and Tables

**Table 1:** Characteristics of the Study Subjects.

	AMD (n = 490)	PCV (n = 419)	Control (n = 1316)	*P* value	
				AMD vs. Control	PCV vs. Control
Gender (male/female)	304/186	294/125	717/599	0.008	<0.001
Mean age ± SD (yrs)					
general	67.5 ± 9.6	64.8 ± 9.7	71.8 ± 5.5	<0.001	<0.001
male	68.1 ± 9.6	65.2 ± 9.7	71.8 ± 5.1	<0.001	<0.001
female	66.5 ± 9.5	63.8 ± 9.4	72.2 ±5.9	<0.001	<0.001
Age range (yrs)					
general	45–89	42–90	60–97	NA	NA
male	45–89	43–90	60–89	NA	NA
female	48–84	42–81	60–97	NA	NA

SD: Standard deviation; NA: not applicable.

**Table 2:** Allelic Association of SNPs in *CFB-SKIV2L-TNXB-FKBPL-NOTCH4* Region with Neovascular AMD and PCV.

SNP	Chromosome	Position	Gene	Minor allele	Minor allele frequency			Allelic association					
					AMD	PCV	Control	AMD-Control		PCV-Control		AMD-PCV	
					(n = 490)	(n = 419)	(n = 1316)	P*	OR* (95%CI)	P*	OR* (95%CI)	P*	OR* (95%CI)
rs541862	6	31949174	*CFB*	G	0.033	0.036	0.043	0.15	0.73(0.48–1.11)	0.41	0.84(0.55–1.27))	0.73	0.91(0.54–1.54)
rs429608	6	31962685	*SKIV2L*	A	0.039	0.063	0.074	**2.2** × **10**^**−4**^	**0.49(0.34**–**0.72)**	0.28	0.84(0.61–1.15)	0.023	0.60(0.39–0.93)
rs12153855	6	32107027	*TNXB*	C	0.075	0.037	0.042	**2.8** × **10**^**−4**^	**1.80(1.31**–**2.48)**	0.51	0.87(0.58–1.31)	**0.001**	**2.10(1.35**–**3.26)**
rs9391734	6	32130206	*FKBPL*	A	0.071	0.030	0.041	**0.001**	**1.76(1.27**–**2.50)**	0.29	0.78(0.49–1.23)	**0.001**	**2.33(1.43**–**3.80)**
rs2071277	6	32203906	*NOTCH4*	G	0.390	0.404	0.382	0.52	1.05(0.90–1.24)	0.24	1.10(0.94–1.30)	0.65	0.96(0.79–1.16)
rs3132946	6	32222251	*NOTCH4*	A	0.003	0.004	0.005	0.14	0.21(0.03–1.63)	0.89	0.71(0.20–2.49)	0.25	0.26(0.03–2.53)
rs800292	1	196673103	*CFH*	T	0.304	0.318	0.416	**1.60** × **10**^**−8**^	**0.62(0.53**–**0.73)**	**5.56** × **10**^**−7**^	**0.65(0.55**–**0.77)**	0.54	0.94(0.76–1.15)
rs11200638	10	122461028	*HTRA1*	A	0.632	0.574	0.445	**8.31** × **10**^**−24**^	**2.31(1.96**–**2.71)**	**3.15** × **10**^**−9**^	**1.65(1.40**–**1.95)**	**0.001**	**1.40(1.14**–**1.72)**

^*^P value and ORs were adjusted for gender; for Bonferroni correction, as 8 SNPs were chosen in this study, a *P* value of less than 0.00625 (0.05/8) was considered statistically significant.

**Table 3:** Results of Association Study by Four Genetic Models.

	SNP	Group	Genotype (n%)			Model	*P*	OR (95% CI)
			AA# (%)	A/B# (%)	BB# (%)			
AMD	rs429608	Control	9 (0.7%)	172 (13.4%)	1106 (85.9%)			
		AMD	0 (0%)	38 (7.7%)	451 (92.3%)	Homo	–	–
						Hetero	**0.002**	**0.54 (0.37**–**0.79)**
						Dominant	**0.001**	**0.52 (0.35**–**0.75)**
						Recessive	–	–
	rs12153855	Control	5 (0.4%)	99 (7.7%)	1185 (91.9%)			
		AMD	3 (0.6%)	65 (13.7%)	406 (85.7%)	Homo	0.32	2.07 (0.49–8.76)
						Hetero	**0.001**	**1.84 (1.31**–**2.60)**
						Dominant	**3.30** × **10**^**−4**^	**1.85 (1.32**–**2.59)**
						Recessive	0.36	1.97 (0.47–8.33)
	rs9391734	Control	5 (0.4%)	94 (7.4%)	1168 (92.2%)			
		AMD	3 (0.7%)	58 (12.8%)	391 (86.5%)	Homo	0.30	2.13 (0.50–8.99)
						Hetero	**0.002**	**1.78 (1.24**–**2.50)**
						Dominant	**0.001**	**1.80 (1.27**–**2.55)**
						Recessive	0.33	2.06 (0.49–8.69)
	rs800292	Control	204 (15.9%)	660 (51.4%)	420 (32.7%)			
		AMD	29 (6.3%)	224 (48.3%)	211 (45.4%)	Homo	**9.12** × **10**^**−9**^	**0.27 (0.17**–**0.41)**
						Hetero	**0.004**	**0.71 (0.56**–**0.89)**
						Dominant	**1.02** × **10**^**−5**^	**0.60 (0.48**–**0.76)**
						Recessive	**3.35** × **10**^**−7**^	**0.33 (0.21**–**0.50)**
	rs11200638	Control	237 (18.8%)	647 (51.4%)	376 (29.8%)			
		AMD	192 (41.8%)	212 (46.2%)	55 (12.0%)	Homo	**2.56** × **10**^**−21**^	**5.69 (3.97**–**8.15)**
						Hetero	**6.94** × **10**^**−7**^	**2.37 (1.69**–**3.33)**
						Dominant	**1.03** × **10**^**−12**^	**3.25 (2.35**–**4.50)**
						Recessive	**1.78** × **10**^**−19**^	**3.03 (2.38**–**3.86)**
PCV	rs800292	Control	204 (15.9%)	660 (51.4%)	420 (32.7%)			
		PCV	35 (8.4%)	194 (46.7%)	186 (44.9%)	Homo	**2.24** × **10**^**−6**^	**0.38 (0.26**–**0.57)**
						Hetero	**0.001**	**0.67 (0.53**–**0.85)**
						Dominant	**1.33** × **10**^**−5**^	**0.60 (0.48**–**0.76)**
						Recessive	**1.14** × **10**^**−4**^	**0.47 (0.32**–**0.69)**
	rs11200638	Control	237 (18.8%)	647 (51.4%)	376 (29.8%)			
		PCV	130 (34.3%)	175 (46.2%)	74 (19.5%)	Homo	**5.86** × **10**^**−9**^	**2.68 (1.92**–**3.73)**
						Hetero	0.047	1.36 (1.00–1.84)
						Dominant	**1.83** × **10**^**−4**^	**1.72 (1.30**–**2.28)**
						Recessive	**2.72** × **10**^**−9**^	**2.18 (1.67**–**2.82)**

^*^P value and ORs were adjusted for gender; ^**#**^A: minor allele, B: major allele.

Genotype (AA/AB/BB) analyses were conducted for the homo model (AA compared with BB), hetero model (AB compared with BB), dominant model (AA+AB compared with BB), and the recessive model (AA compared with AB+BB).

**Table 4:** Association of rs12153855 and rs9391734 with Neovascular AMD in Stratification of rs11200638, rs800292 and rs429608 Genotypes.

SNP	Genotypes	Rs12153855					RS9391734				
		CC	CT	TT	Allelic*P*	OR (95% CI)	AA	AG	GG	Allelic*P*	OR (95% CI)
	**AA+AG**										
	Case	3	58	342	**1.5** × **10**^**−5**^	**2.14 (1.50**–**3.04)**	3	57	322	**8.0** × **10**^**−6**^	**2.21 (1.55**–**3.15)**
Rs11200638	Control	3	62	812			3	61	792		
	**GG**										
	Case	0	5	50	0.77	0.87 (0.33–2.25)	0	5	47	0.98	1.01 (0.39–2.64)
	Control	2	35	338			2	31	335		
	**CC+CT**										
	Case	3	58	372	**6.5** × **10**^**−4**^	**1.75 (1.27**–**2.44)**	3	50	349	**0.003**	**1.67 (1.18**–**2.36)**
Rs800292	Control	4	85	983			4	81	954		
	**TT**										
	Case	0	4	25	0.29	1.81 (0.59–5.63)	0	5	24	0.06	2.64 (0.91–7.63)
	Control	1	14	189			1	12	190		
	**GG+AG**										
	Case	3	65	406	**1.2** × **10**^**−4**^	**1.82 (1.33**–**2.47)**	3	58	391	**4.4** × **10**^**−4**^	**1.77 (1.28**–**2.44)**
Rs4290608	Control	5	99	1176			5	94	1159		
	**AA**										
	Case	0	0	0	NA	NA	0	0	0	NA	NA
	Control	0	0	9			0	0	9		

The stratification of rs11200638, rs800292 and rs429608 were under the dominant model with the risk allele as reference. The genetic effect of rs12153855 and rs9391734 were evaluated under the allelic model in the context of rs11200638, rs800292 and rs429608. For Bonferroni correction, as we analyzed 2 SNPs (rs12153855 and rs9391734) under 6 strata (2 strata for each of rs11200638, rs800292 and rs429608), a *P* value less than 0.0042 [0.05/ (2 SNPs × 6 strata)] was considered statistically significant.

**Table 5:** Multiple Logistic Regression Analysis of the *CFB-SKIV2L-TNXB-FKBPL-NOTCH4* Region*, CFH* rs800292*, HTRA1* rs11200638 in the Association with Neovascular AMD and PCV.

Variable	AMD		PCV	
	*P*	OR	*P*	OR
*CFB* rs541862	0.60	1.19 (0.61–2.34)	0.37	0.74 (0.38–1.43)
*SKIV2L* rs429608	**0.016**	**0.49 (0.27**–**0.87)**	0.60	0.87 (0.54–1.45)
*TNXB* rs12153855	0.21	1.53 (0.79–2.98)	0.30	0.63 (0.26–1.52)
*FKBPL* rs9391734	0.82	1.09 (0.55–2.14)	0.98	1.01 (0.42–2.45)
*NOTCH4* rs2071277	0.48	0.93 (0.77–1.13)	0.21	1.13 (0.93–1.37)
*NOTCH4* rs3132946	0.23	0.28 (0.04–2.25)	0.98	0.98 (0.26–3.66)
*CFH* rs800292	**4.08** × **10**^**−14**^	**0.48 (0.39**–**0.58)**	**1.32** × **10**^**−10**^	**0.52 (0.43**–**0.64)**
*HTRA1* rs11200638	**8.94** × **10**^**−29**^	**2.85 (2.37**–**3.43)**	**6.69** × **10**^**−14**^	**2.05 (1.70**–**2.47)**

For each SNP, the minor alleles were taken as reference. Multiple logistic regression analysis was conducted under the allelic model. It is statistically significant when *P* < 0.05.
