# Supplementary material for: Associations of 6p21.3 Region with Age-related Macular Degeneration and Polypoidal Choroidal Vasculopathy
Source: Sci Rep. 2016 Feb 10;6:20914. doi: 10.1038/srep20914 (PMC4748259; doi:10.1038/srep20914)
Supplement: Supplementary Information [file srep20914-s1.pdf]

Supplementary Material

Associations of 6p21.3 Region with Age-related Macular Degeneration and Polypoidal Choroidal Vasculopathy

Zimeng Ye [1,2 #], Ping Shuai [1,3 #], Yaru Zhai [1 #], Fang Li [1,4 #], Lingxi Jiang [1], Fang Lu [1,2], Feng Wen [5], Lulin Huang [1], Dingding Zhang [1,3], Xiaoqi Liu [1], Ying Lin [1], Huaichao Luo [1,6], Houbin Zhang [1], Xianjun Zhu [1,2], Zhengzheng Wu [1,4], Zhenglin Yang [1,2], Bo Gong [1 *], Yi Shi [1,2 *]

[1] Sichuan Provincial Key Laboratory for Human Disease Gene Study, School of Medicine, Sichuan Academy of Medical Sciences & Sichuan Provincial People's Hospital, University of Electronic Science and Technology of China, Chengdu, China; [2] College of Life Science and Engineering, Southwest Jiaotong University, Chengdu, China; [3] Health Management Center, Sichuan Provincial People's Hospital, Chengdu, China; [4]Department of ophthalmology, Sichuan Provincial People's Hospital, Chengdu, China; [5] Zhongshan Ophthalmic Center, Guangzhou, China; [6] Clinical Medicine Department, Luzhou Medical College, Luzhou, China.

# These authors contributed equally.

***Correspondence:**

Yi Shi (yshi@uestc.edu.cn) and Bo Gong (gongbo2007@hotmail.com), Sichuan Provincial Key Laboratory for Human Disease Gene Study, School of Medicine, Sichuan Academy of Medical Sciences & Sichuan Provincial People's Hospital, University of Electronic Science and Technology of China, 32 the First Ring Road West 2, Chengdu, Sichuan 610072, China; Tel.: +86 2887393375; Fax: +86 2887393548.

Contents:

Supplementary Figure 1

Supplementary Table 1, 2, 3, 4

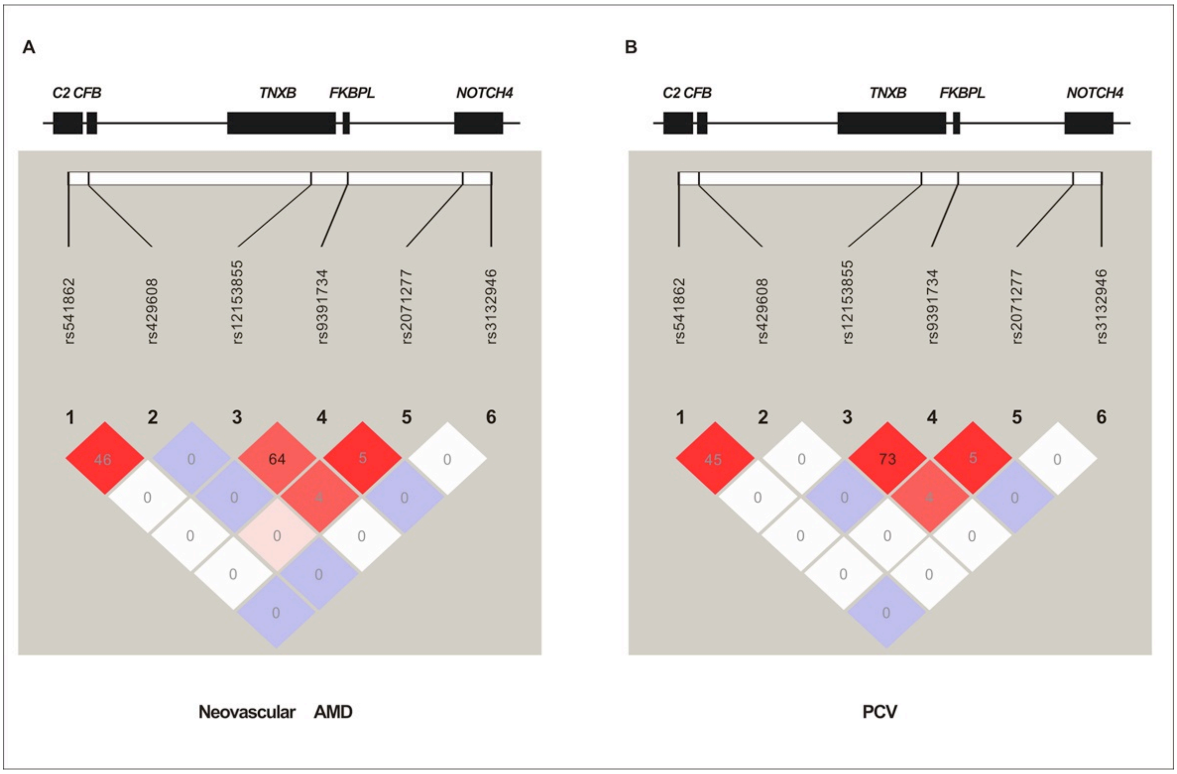

Supplementary Figure1:

Linkage disequilibrium (LD) structure across *CFB-SKIV2L-TNXB-FKBPL-NOTCH4* region (r^2 values shown). A. LD was measured byusing combined AMD case and normal controls data. The physical position of each SNP is shown in the upper diagram. Each box provides estimated statistics of the coefficient of determination (r^2), with darker shades representing stronger LD. **B.** LD was measured using combined PCV case and normal controls data.

Supplementary Table 1. Hardy-Weinburg equilibrium of each SNP in this study

SNP	Chromosome	Position	Gene	HWE-Control	HWE-AMD	HWE-PCV
**rs541862**	6	31949174	*CFB*	0.07	0.50	0.53
**rs429608**	6	31962685	*SKIV2L*	0.42	0.37	0.58
**rs12153855**	6	32107027	*TNXB*	0.06	0.82	0.56
**rs9391734**	6	32130206	*FKBPL*	0.05	0.60	0.29
**rs2071277**	6	32203906	*NOTCH4*	0.49	0.19	0.71
**rs3132946**	6	32222251	*NOTCH4*	0.84	0.94	0.94
**rs800292**	1	196673103	*CFH*	0.05	0.002	0.11
**rs11200638**	10	122461028	*HTRA1*	0.16	0.76	0.27

Supplementary Table 2. Associations of 4 Genetic Models of SNPs in *CFB-SKIV2L-TNXB-FKBPL-NOTCH4* region with AMD

SNP	Group	Genotype (n%) AA# （%）	A/B# （%）	BB# （%）	Model	*P**	OR* (95% CI)
rs541862	Control	5 (0.4%)	100 (7.7%)	1187 (91.9%)			
	AMD	1 (0.2%)	30 (6.2%)	450 (93.6%)	Homo	0.59	0.56 (0.07-4.82)
					Hetero	0.19	0.74 (0.47-1.15)
					Dominant	0.21	0.76 (0.49-1.17)
					Recessive	0.61	0.57 (0.07-4.90)
rs429608	Control	9 (0.7%)	172 (13.4%)	1106 (85.9%)			
	AMD	0 (0%)	38 (7.7%)	451 (92.3%)	Homo	--	--
					Hetero	**0.002**	**0.54 (0.37-0.79)**
					Dominant	**0.001**	**0.52 (0.35-0.75)**
					Recessive	--	--
rs12153855	Control	5 (0.4%)	99 (7.7%)	1185 (91.9%)			
	AMD	3 (0.6%)	65 (13.7%)	406 (85.7%)	Homo	0.32	2.07 (0.49-8.76)
					Hetero	**0.001**	**1.84 (1.31-2.60)**
					Dominant	**3.30×10^{-4}**	**1.85 (1.32-2.59)**
					Recessive	0.36	1.97 (0.47-8.33)
rs9391734	Control	5 (0.4%)	94 (7.4%)	1168 (92.2%)			
	AMD	3 (0.7%)	58 (12.8%)	391 (86.5%)	Homo	0.30	2.13 (0.50-8.99)
					Hetero	**0.002**	**1.78 (1.24-2.50)**
					Dominant	**0.001**	**1.80 (1.27-2.55)**
					Recessive	0.33	2.06 (0.49-8.69)
rs2071277	Control	179 (14.1%)	611 (48.1%)	480 (37.8%)			
	AMD	65 (13.8%)	238 (50.4%)	169 (35.8%)	Homo	0.63	1.08 (0.77-1.53)
					Hetero	0.41	1.11 (0.87-1.41)
					Dominant	0.39	1.10 (0.88-1.39)
					Recessive	0.89	1.02 (0.75-1.40)
rs3132946	Control	0 (0%)	14 (2.8%)	1277 (97.2%)			
	AMD	0 (0%)	3 (0.6%)	473 (99.4%)	Homo	--	--
					Hetero	0.14	0.21 (0.03-1.61)
					Dominant	0.14	0.21 (0.03-1.61)
					Recessive	--	--
rs800292	Control	204 (15.9%)	660 (51.4%)	420 (32.7%)			
	AMD	29 (6.3%)	224 (48.3%)	211 (45.4%)	Homo	**9.12×10^{-9}**	**0.27 (0.17-0.41)**
					Hetero	**0.004**	**0.71 (0.56-0.89)**
					Dominant	**1.02×10^{-5}**	**0.60 (0.48-0.76)**
					Recessive	**3.35×10^{-7}**	**0.33 (0.21-0.50)**
rs11200638	Control	237 (18.8%)	647 (51.4%)	376 (29.8%)			
	AMD	192 (41.8%)	212 (46.2%)	55 (12.0%)	Homo	**2.56×10^{-21}**	**5.69 (3.97-8.15)**
					Hetero	**6.94×10^{-7}**	**2.37 (1.69-3.33)**
					Dominant	**1.03×10^{-12}**	**3.25 (2.35-4.50)**
					Recessive	**1.78×10^{-19}**	**3.03 (2.38-3.86)**

**P* value and ORs were adjusted by gender; # A: minor allele, B: major allele.

Genotype (AA/AB/BB) analyses were conducted for the homo model (AA compared with BB), hetero model (AB compared with BB), dominant model (AA+AB compared with BB)， and the recessive model (AA compared with AB+BB).

Supplementary Table 3. Associations of 4 Genetic Models of SNPs in *CFB-SKIV2L-TNXB-FKBPL-NOTCH4* region with PCV

SNP	Group	Genotype (n%)			Model	*P**	OR* (95% CI)
		AA# （%）	A/B# （%）	BB# （%）			
rs541862	Control	5 (0.4%)	100 (7.7%)	1187 (91.9%)			
	PCV	1 (0.2%)	28 (6.8%)	381 (93.0%)	Homo	0.66	0.62 (0.67-5.35)
					Hetero	0.54	0.87 (0.56-1.35)
					Dominant	0.49	0.86 (0.56-1.32)
					Recessive	0.67	0.63 (0.07-5.40)
rs429608	Control	9 (0.7%)	172 (13.4%)	1106 (85.9%)			
	PCV	1 (0.2%)	51 (12.2%)	367 (87.6%)	Homo	0.28	0.33 (0.04-2.65)
					Hetero	0.51	0.89 (0.63-1.24)
					Dominant	0.39	0.87 (0.62-1.20)
					Recessive	0.67	0.34 (0.04-2.69)
rs12153855	Control	5 (0.4%)	99 (7.7%)	1185 (91.9%)			
	PCV	1 (0.2%)	29 (7%)	385 (92.8%)	Homo	0.66	0.61 (0.07-5.28)
					Hetero	0.64	0.90 (0.59-1.38)
					Dominant	0.58	0.89 (0.58-1.35)
					Recessive	0.66	0.62 (0.07-5.32)
rs9391734	Control	5 (0.4%)	94 (7.4%)	1168 (92.2%)			
	PCV	1 (0.3%)	22 (5.8%)	356 (93.9%)	Homo	0.69	0.66 (0.07-5.63)
					Hetero	0.27	0.76 (0.47-1.24)
					Dominant	0.25	0.76 (0.47-1.22)
					Recessive	0.71	0.67 (0.08-5.73)
rs2071277	Control	179 (14.1%)	611 (48.1%)	480 (37.8%)			
	PCV	66 (15.9%)	204 (49.0%)	146 (35.1%)	Homo	0.26	1.21 (0.86-1.69)
					Hetero	0.45	1.09 (0.86-1.40)
					Dominant	0.32	1.12 (0.89-1.41)
					Recessive	0.37	1.14 (0.84-1.56)
rs3132946	Control	0 (0%)	14 (2.8%)	1277 (97.2%)			
	PCV	0 (0%)	3 (0.7%)	400 (99.3%)	Homo	--	--
					Hetero	0.59	0.71 (0.20-2.49)
					Dominant	0.59	0.71 (0.20-2.49)
					Recessive	--	--
rs800292	Control	204 (15.9%)	660 (51.4%)	420 (32.7%)			
	PCV	35 (8.4%)	194 (46.7%)	186 (44.9%)	Homo	**2.24×10^{-6}**	**0.38 (0.26-0.57)**
					Hetero	**0.001**	**0.67 (0.53-0.85)**
					Dominant	**1.33×10^{-5}**	**0.60 (0.48-0.76)**
					Recessive	**1.14×10^{-4}**	**0.47 (0.32-0.69)**
rs11200638	Control	237 (18.8%)	647 (51.4%)	376 (29.8%)			
	PCV	130 (34.3%)	175 (46.2%)	74 (19.5%)	Homo	**5.86×10^{-9}**	**2.68 (1.92-3.73)**
					Hetero	0.047	1.36 (1.00-1.84)
					Dominant	**1.83×10^{-4}**	**1.72 (1.30-2.28)**
					Recessive	**2.72×10^{-9}**	**2.18 (1.67-2.82)**

**P* value and ORs were adjusted by gender; # A: minor allele, B: major allele.

Genotype (AA/AB/BB) analyses were conducted for the homo model (AA compared with BB), hetero model (AB compared with BB), dominant model (AA+AB compared with BB)， and the recessive model (AA compared with AB+BB).

Supplementary Table 4. Re-evaluation of the Association of rs12661281 with Neovascular AMD and PCV

	Group	Genotype* (n%)			MAF	Model	*P* Value	OR (95% CI)
		AA	A/T	TT				
**nAMD**	**Control**	7 (0.7)	184 (18.1)	823 (81.2)	0.098			
	**Patient**	16 (2.8)	108 (18.9)	447 (78.3)	0.123	**Allelic**	**0.028**	**1.29 (1.02-1.62)**
						**Homo**	**0.00066**	**4.21 (1.72-10.31)**
						**Hetero**	0.56	1.08 (0.83-1.40)
						**Dominant**	0.17	1.20 (0.93-1.54)
						**Recessive**	**0.00073**	**4.15 (1.70-10.14)**
**PCV**	**Control**	7 (0.7)	184 (18.1)	823 (81.2)	0.098			
	**Patient**	4 (1.0)	73 (18.2)	324 (80.8)	0.101	**Allelic**	0.79	1.03 (0.79-1.36)
						**Homo**	0.55	1.45 (0.42-4.99)
						**Hetero**	0.96	1.01 (0.74-1.36)
						**Dominant**	0.87	1.02 (0.76-1.37)
						**Recessive**	0.55	1.45 (0.42-4.98)
**nAMD +PCV**	**Control**	7 (0.7)	184 (18.1)	823 (81.2)	0.098			
	**Patient**	20 (2.1)	181 (18.6)	771 (79.3)	0.112	**Allelic**	0.12	1.17 (0.97-1.45)
						**Homo**	**0.0081**	**3.04 (1.28-7.25)**
						**Hetero**	0.67	1.05 (0.84-1.32)
						**Dominant**	0.30	1.12 (0.90-1.40)
						**Recessive**	**0.0085**	**3.02 (1.27-7.18)**

Genotypes were gained from the previous replication study in the GAMA Consortium research (ref. 27). The characteristics of the Sichuan replication cohort were reported in ref.27.
